# Telemedicine in Peru: origin, implementation, pandemic escalation, and prospects in the new normal

**DOI:** 10.1093/oodh/oqae002

**Published:** 2024-01-12

**Authors:** Gareth H Rees, Felipe Peralta

**Affiliations:** Department of Marketing and Administration, ESAN University, Alonso de Molina 1652, Monterrico Chico, Lima 33, Peru; Department of Preventive Medicine and Public Health, Universidad Nacional Mayor de San Marcos (UNMSM), Lima 15081, Peru

**Keywords:** Digital Health, Telemedicine, Peru, Universal Health Coverage, Service development

## Abstract

For many countries telemedicine was speedily adopted as a result of the COVID-19 pandemic, though for some countries telemedicine may have been implemented in a context of limited regulations or few plans or strategies to scale quickly. This article recounts how telemedicine was developed in Peru as a measure to support the country's Universal Health Coverage and service access to rural and locations with low workforce numbers and its deployment. From a range of data, we find that Peru's development of telehealth began before the pandemic, which by 2020 was sufficient to be able to foster a rapid and wider deployment and while the telemedicine service volumes quickly grew from the pandemic onset, these numbers then begin to reduce suggesting that telemedicine was considered more as a pandemic emergency measure rather than a change to the mix of health provision. From these data we offer two lessons, (i) that Peru's preparedness in terms of telemedicine policy and regulation were helpful to rapidly expand telemedicine at a time of necessity and (ii) that due to this investment and with a better understanding, Peru now has a short-run window of opportunity for the Peruvian Government to continue its regulatory development and investment to further deploy telemedicine services as a UHC improvement measure and to better align the health system to the country’s health needs.

## INTRODUCTION

The Covid-19 pandemic affected how treatments and exchanges between patients and health workers were undertaken, due to its transmission risks [1]. In response, many countries applied Information and Communication Technology (ICT), to advance their telehealth programs and to create physical distances in clinical, administrative and social interactions between people in the health system [2, 3]. Telemedicine has been defined as the use of ICTs such as the telephone, video conferencing or other secure messaging that enable synchronous or asynchronous consultations to overcome geographical and functional distances and provides advantages, such as convenience, cost savings, optimizing multidisciplinary visits and real-time communication with colleagues [4], specifically referring to clinical services [5]. Thus, telemedicine was seen by many countries as an attractive option early in the pandemic to continue medical interactions and so was widely and speedily adopted [6].

However, this rapid increase in telemedicine’s use was largely based on knowledge from situations quite different from those experienced in the pandemic [1] and much of what we know of its benefits and barriers tends to be based mainly on developed country experiences, with less low- and middle-income country (LMIC) evidence available [7]. These benefits relate to delivering access to quality health care services and outcomes efficiently and cost-effectively [4], providing access to specialist services and reducing overcrowding of emergency departments and hospitals [8] and providing access to health services to those with economic, social and geographical disadvantages [9]. In Latin America during the pandemic, telemedicine contributed to the continuity of care for patients particularly those with chronic non-communicable diseases and mental health conditions, while pre pandemic, it produced cost savings in the region's publicly funded services as more telemedicine is provided through these services than through those that are privately owned and operated [10]. However, weak digital health legal frameworks in many Latin American countries are cited as a telemedicine barrier, where licensing and registration, unclear or conflicting regulations and poor oversight are considered to impede its adoption [10]. Other limitations related to telemedicine are reduced performances for patient physical examinations, the possibilities for technical difficulties, data security breaches [4], health workforce and patient technology skill levels and training needs, patient preferences for in-person consultations [11] and patient quality perceptions [12]. So, while telemedicine adoption may be rising, unequal access and use exists across clinical conditions, age groups, geographical locations and for those with limited access to in-person care; all of which suggests that health care organizations and governments have more work to do to reduce these disparities [13].

The Latin American LMIC Peru was significantly affected by Covid-19 and experienced the world's highest per capita death rate of 5.2% or 659 per 100,000 population [14]. This situation occurred despite an early implementation of public health measures aimed to reduce contact between people and the spread of the virus [15, 16] that included a remote work policy for its health personnel [17]. Peru's high levels of inequality and economic informality have been suggested as a contributor the pandemic’s outcomes as informal workers are less likely to follow quarantine and social distancing measures and have poorer access to health services [18, 19]. While the central government's pandemic response was rapid, its inability to project its pandemic response measures uniformly throughout the country left many rural areas paralysed or resorting to improvised responses [20]. During this time, Peru also experienced some of the aforementioned barriers to telemedicine adoption such increased needs for training, patient consultation preferences and connectivity issues [12]. With Peru's COVID-19 emergency measures concluding on 27 October 2022 [21], Johns Hopkins University ceasing its global COVID-19 data collection and curation on 10 March 2023 [14] and the WHO’s declaration that that COVID-19 is no longer a global health emergency on 5 May 2023 [22], we consider it an appropriate time to review how telemedicine has developed in Peru, its contribution to the country’s pandemic response and consideration of its future contribution to Peru's health system development.

## MATERIALS AND METHODS

The article was developed using data collected from a rapid literature search using primarily Google scholar and the main Google website using key words such as ‘Peru’, ‘Telemedicine’ and ‘Pandemic’ and then from applying the snowball sampling technique [23]. From these search actions, a range of peer-reviewed literature were identified along with relevant Peruvian Government strategy documents, multilateral agency reports and website postings. The telemedicine usage data used in this article was sourced from the Minsa transparency portal [24] through the required request forms, asking for telemedicine data for the years 2019 to 2022 the type of telemedicine service in total and for each of the 25 operational regions of Peru’s publicly funded health system. These data were supplied in excel files sourced from the Minsa database and presented as coded using the 2020 telemedicine type categories as defined by 2020’s Legislative Decree No.1490.

## RESULTS

### Peru's health system

The upper-middle income country of Peru consists of 32 million people that are organized into 25 provincial regions, many of which (80%) live in urban settings [25]. Poverty in Peru has experienced significant reductions over the past twenty years. However, much of the last decade's advances were erased in the first year of the pandemic, with the 2021 rate of population in a situation of monetary poverty recorded as 25.9%, a return to the rate of 2012 and those experiencing extreme poverty (spending less than S/201, about US$ 54^1^, per month per person) returning to the 2015 rate of 4.1% [26]. Peruvians experiencing high levels of poverty tend to reside in rural rather than urban areas and in the Andean and Amazonian regions [26].

Peru spends 4.8% of GDP on health care, its health system receives 51.6% from public expenditure, with 38.4% of the total health expenditure being provided as out of pocket expenses [27]. Peru has made significant gains on health financing and UHC, with the percentage of uninsured reducing from 63% in 2004 to 13% in 2018 [28] and Peruvians with at least one insurance affiliation recorded as 95% in 2020 [29]. This progress towards higher insurance affiliation rates and UHC was significantly influenced by the introduction of the Comprehensive Health Insurance scheme, Seguro Integral de Salud (SIS), in 2002: a service that extends health-care services to the country’s poor and uninsured populations [28]. This was fortified by the 2019 declaration that the SIS system will enrol anyone without health insurance providing cover for the residual four million Peruvian estimated not to be covered at that time [30]. Thus, access to insurance affiliation may not be the most significant barrier to health access in Peru. Rather, the prevalence of long wait times and service locations are identified as significant barriers to be overcome for those the not able to access care [31].

Low-income Peruvians receive access to health services from the SIS scheme that provides services for about 60% of the population, with formal workers covered through payments they make as affiliates to the Social Health Insurance Scheme, with similar worker-based schemes existing for each branch of the armed forces and police, covering about 30% of the population. These insurance schemes are complemented by a small private insurance market providing health cover for the working non-affiliated and the more well-off remaining 10% [27]. However, the existence of multiple health insurers and the services that they fund and or operate has led to the Peruvian health system being described as ‘often working with a high degree of overlap and little coordination’ [32 p.16]. Despite this fragmented system, Peruvian life expectancy has been steadily improving along with sustained reductions in mortality and fertility rates, which are attributed to the country’s recent economic performance and poverty reduction efforts [27] and are producing a population pyramid transformed from a wide base to one exhibiting a middle bulge [29]. This changing population mix has had the effect of placing increasing pressure on a health system that has not adapted to the epidemiological transition of growing chronic disease incidence, constant contagious disease prevalence and persistent high accident and injury rates [29].

Therefore, as the attainment of UHC has been stalling in recent years [33], the Peruvian government continues to seek to capture the benefits of telemedicine by its inclusion in the country's strategic health plan along with other Health Information System Management (HSIM) initiatives such as strengthening of electronic health records, digital and ITC infrastructures [34]. More detailed HSIM performances are contained in the Ministry's Digital Health Sector Agenda, which outlines the scope and actions to promote telemedicine through the provision of a single platform, the provision of online consultation reservation availability for patients and through system standardization and integration, that are measured by assigned metrics [35]. While these plans are an important measure, the World Bank reports that there has been low performance of previously planned initiatives; for instance, only 15% of primary care health establishments have access to electronic patient records [36].

### Peru and telemedicine

#### Peru's telemedicine development

Telemedicine began in Peru indirectly, through the push to improve the population’s access to ICTs through broadband and cellular telephony. Initiated through the 2001 Supreme Decree No. 066-2001-PCM that promoted the expansion of internet access in Peru. This legislation considered the impacts of not just expanding Internet access but included service expansion advantages, such as online education, teleworking and telehealth. As there have been a few previously published perspective articles on Peru's telemedicine that deal with the development of Peru's regulatory framework in depth (see Alverez Risco et al. [37] and Curioso et al. [38]), we will not repeat this. But we do note that this regulatory development sequencing throughout the second decade of this century was a significant facilitator for the adoption of telemedicine in the pandemic. In addition, the authors comment on the importance of also improving health-information systems, health professionals training in digital skills and the support of health and data-science management, and a cultural change in health services framing [38], the use of telemedicine for chronic disease monitoring and care, as well as the ongoing issue of access to not only health services, but to the internet as well [37] as key issues confronting Peru’s telemedicine development.

Around the time the first sets of telemedicine regulations were being implemented (in and around 2008), Peru had a cell phone subscription rate per 100 people of 73.35, and 30.57% of its population using the internet, while at the onset of the pandemic its 2020 cell phone subscription rate had increased to 133.44 and the percentage of population using the internet rose to 65.25% [39], significantly enlarging the potential users of telemedicine services. However, Peruvian internet prices remain high compared with other Latin American countries [40], suggesting that while its internet and mobile broadband network coverage may have improved, there is likely an ongoing access barrier for poor and marginalized groups in the country. Nevertheless, this issue was recognized early on in Peru’s the regulatory development, where the health system development project PARSALUD-II, developed and initiated a model of teleconsultations and teletraining was for maternal health services in the Andean region of Huánuco and Amazonian regions of Amazonas and Ucayali; locations with poor access to neonatal specialists [41]. The PARSALUD-II project revealed that it was possible to prioritize and support interventions that aimed to narrow health gaps for citizens in rural and hard to reach areas using the features of telemedicine, and it became the starting point for developing a national system of teleconsultation registration. This led into a second round of regulatory development that relied on stakeholder consultations on how telehealth could be applied as a strategy to improve access to medical specialists in poorly resourced geographical areas, to strengthen health workforce capacity, to contribute to UHC goals and to improve the health of people across the country, while recognizing some of the implementation difficulties associated with these goals. Thus, the regulatory push in the later part of the 2020s led to program-based strategies and the creation of a responsible unit in Minsa, the Directorate of Telemedicine (DoT). This unit's role is to be responsible for formulating and implementing standards, plans, strategies and guidelines for the implementation of telemedicine in the health sector, as well as to provide remote services in the components of promotion, prevention, diagnosis, recovery or rehabilitation, and to carry out their follow-up and monitoring. The DoT delivers on these objectives through a portfolio of services offered in health facilities and the four activities of telemedicine for diagnosis defined in Legislative Decree No.1490: being teleconsultation, teleinterconsultation, telemonitoring and teleorientation [42].

Shortly after, the Covid-19 pandemic occurred and due to the existing regulatory framework the Government was able to approve the National Telehealth Plan 2020–2023 in December 2020 and promulgate a range of emergency administrative regulations. These provided for the aforementioned more detailed definitions of telemedicine services, as well as regulations for e-prescriptions and health information sharing (Legislative Decree No.1490), budgetary support for telehealth initiatives (Law N° 31 365—Public Sector Budget Law for 2022) and locality extensions for telemedicine services (Ministerial Resolution N° 081–2022-MINSA). However, while Legislative Decree No.1490 greenlighted the wider use of e-prescriptions, it has been reported that there is little uptake data by physicians and pharmacists [37].

#### Peru's telemedicine deployment and utilisation

**Figure 1:**
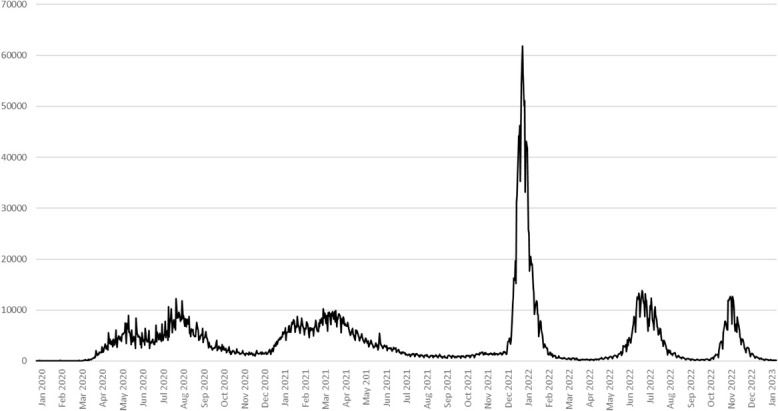
Covid-19 case rates Peru 2020 to 2023. Source: Minsa 2024, https://www.dge.gob.pe/covid19.html

**Figure 2:**
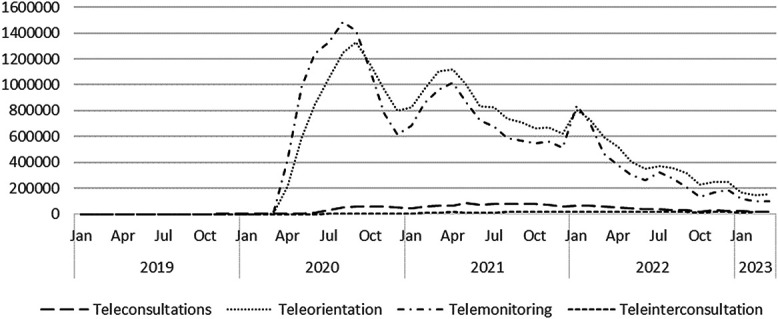
Use of publicly funded telemedicine services in Peru 2019 to 2023. Source: Sistema de Salud Asistencial, Minsa 2023

Peru's publicly funded telemedicine use was relatively small before the pandemic with a total of 11 388 interactions recorded in 2019. However, this increased substantially as the aforementioned telemedicine regulations took effect. To contextualize this growth, Fig. 1 shows the waves of COVID-19 cases experienced in Peru, while Fig. 2 shows the use of the four telemedicine activities in the country from January 2019 to March 2023. These data are reflective of the increased mobile data and cable-based broadband activity across Peru during the pandemic period, where in the pandemic's first year, mobile telephone services grew 15.52% and fixed telephony service grew by 2.77% and bundled internet and television service subscriptions grew by 14.72% [43].

Figure 2 reveals the rapid rise of the use of publicly funded telemedicine services, which corresponds to the early waves of infection, tailing off as the pandemic progresses. Alvarez-Risco et al. reveal that throughout this time Peruvian telemedicine services were used for a range of patient monitoring and care continuity for chronic diseases such as diabetes, cardiovascular and tuberculosis, as well as for those suffering from chronic pain [37]. Therefore, it is not surprising that most of the publicly funded telemedicine use is reflected by two activities, teleorientation and telemonitoring, which relate to ongoing monitoring of patient welfare and disease progression or impacts. Telemonitoring may also have experienced a higher volume due to the transmission of test results and the advice given based on results, and while e-prescription frequency is not known, the volume of telemonitoring infers that there may have been some increase in digital prescribing. Average monthly teleconsultations are lower in 2020 as facilities and infrastructures are instituted to reach 3232, though later months are more than double this. In response to reduced access to onsite facilities, the 2021 monthly average increases to be 70 463, but for 2022 the monthly average then falls to 42 932 (a close to 40% reduction) and continues to reduce into 2023 with the average number of monthly teleconsultations for the first 3 months at 21910.

This pattern indicates that perhaps telemedicine services were viewed by the Peruvian public and health workforce as solely an emergency measure, rather than as a mainstream model of care. As such, the results of the pandemic's pattern of publicly funded telemedicine service use, while promising, requires further study on the reasons for this fluctuation in use, its service quality and health effects and to better understand telemedicine’s benefits [37].

Table 1 provides some insight into the total publicly funded telemedicine activities per region for the full years 2019 to 2022, along with the regions’ SIS affiliated populations in December 2022 and the 2022 regional poverty rates.

**Table 1:** Annual Regional Publicly Funded Telemedicine Interactions, SIS affiliations and Poverty rates (Total for full years 2019–2022)

Region	Tele Consultations*	Tele Inter consultations*	Tele Monitoring*	Tele Orientation*	Total Tele Interactions*	SIS affiliated population^#^	Regional Poverty (%)^
Amazonas	5078	1108	191 243	258 020	455 449	450 801	27.92
Ancash	67 783	20 092	1 403 968	1 273 275	2 765 118	969 837	17.55
Apurimac	69 993	15 867	800 456	1 012 155	1 898 471	404 543	10.74
Arequipa	102 049	6720	1 399 435	926 901	2 435 105	953 321	10.56
Ayacucho	17 613	22 775	641 901	639 499	1 321 788	607 413	16.89
Cajamarca	44 481	20 385	738 496	706 146	1 509 508	1 409 839	14.11
Callao	41 777	4134	1 188 101	760 570	1 994 582	697 149	8.89
Cusco	21 040	16 207	715 398	708 373	1 461 018	1 165 959	12.26
Huancavelica	26 117	30 205	629 508	258 993	944 823	362 269	12.90
Huanuco	25 392	17 321	761 326	1 187 076	1 991 115	755 485	23.67
Ica	45 703	4317	537 186	615 265	1 202 471	590 162	11.86
Junin	71 114	45 072	1 793 652	1 605 291	3 515 129	1 132 522	23.48
La Libertad	54 970	12 488	954 782	1 882 429	2 904 669	1 508 469	10.73
Lambayeque	51 613	6305	911 798	1 256 898	2 226 614	987 289	10.25
Lima	819 859	92 149	5 447 477	6 640 176	12 999 661	7 056 552	17.38
Loreto	3037	2721	119 695	66 386	191 839	1 045 097	56.24
Madre De Dios	2995	1132	194 694	187 650	386 471	167 101	21.08
Moquegua	11 124	906	208 311	187 897	408 238	119 663	10.19
Pasco	9474	10 803	530 448	407 380	958 105	218 424	21.90
Piura	100 509	40 415	962 585	830 309	1 933 818	1 572 751	22.61
Puno	15 260	8007	599 638	623 632	1 246 537	1 087 469	20.51
San Martin	28 811	9558	228 809	238 599	505 777	862 366	30.94
Tacna	17 349	5889	485 907	440 469	949 614	281 892	5.99
Tumbes	10 388	329	165 471	141 744	317 932	200 071	21.18
Ucayali	4251	5570	269 031	150 302	429 154	567 326	42.90

^*^Sistema de Salud Asistencial Minsa 2023

# Regional SIS affiliated population (December 2022) https://www.gob.pe/institucion/sis/informes-publicaciones/4148587-estadistica-de-asegurados-por-mes-diciembre-2022

^ INEI Regional poverty rates 2022 https://www.inei.gob.pe/media/MenuRecursivo/indices_tematicos/cd4_23.xlsx

The increase in Peruvian telemedicine activity over the pandemic is also reflected in the introduction other digital health aids such as mobile applications and online monitoring programs, of which a number were developed and introduced for oncology, tuberculosis, and diabetes patients from 2020 through 2021 [37]. With this increase in digital health activity, a directive for online consultations and management of chronic disease patients was developed and made available [44]. However, it has also been suggested that the regulation of digital health in Peru needs more attention, due to issues such as security and privacy, quality of information and the level of results or benefits they provide [38].

To access telemedicine activities the Ministry of Health now uses the ‘Teleatiendo’ health information system [45]. It was devised relatively early in the pandemic as an immediate response to monitor COVID-19 diagnosed patients to reduce further community spread. It was later available to patients with some vulnerability to the virus, such as people with disabilities and those that suffer from chronic diseases such as hypertension, diabetes, cardiovascular diseases, Parkinson's, Alzheimer's or patients with cancer treatments and enables access to the range of the state's telemedicine activities [46].

In line with the rise of telemedicine services during the pandemic, the public-sector health workforce was provided access to training and coordination through online means. These activities included virtual national workshops and international Congresses to promote a space for dialogue and updating telehealth knowledge for all health personnel, ICT support personnel and medical professionals associated with Peru's telehealth areas [47]. Additionally, a Diploma in Telemedicine Management and higher qualifications have been developed and offered to health care workers [48]. The diploma is particularly relevant to telemedicine performance measures, as at least one person in a facility providing telemedicine should be trained to this level [49].

To enable continued activities of publicly funded Telehealth the Public Sector Budget Laws for Fiscal Years 2021 and 2022 (Article 33.1 of Law No. 31084 and Law No. 31365 respectively) appropriated funds to be applied to the strategic lines of the Telehealth Plan. For telemedicine, these funds allowed an extension project that identified 678 primary care sites across the regions of Peru to increase the number of publicly funded telemedicine sites [50]. The project assessed needs, sourced and supplied equipment to the primary care centres completing its work in early 2023 [51].

## DISCUSSION

We have outlined Peru’s telemedicine interventions up to during and just after the pandemic. As such, we have noted Peru’s long policy and regulatory development gestation, incorporating the intention to improve service access for those in poorly resourced areas as key part of telemedicine’s development and inception. This policy effort created a series of legal frameworks, plans, and clarified responsibilities were the basis for a rapid increase of telemedicine services in response to the COVID-19 pandemic. The usage data we present reveals that telemedicine volumes quickly grew but started to reduce following the second wave of COVID-19 infections in mid-2021. However, it has been reported that large proportion of these interactions appear to be associated with urban rather than in the more rural locations where Peru's poor and disadvantaged tend to reside [37]. However, the regional distribution data show that some poorer districts in fact have high utilizations rates, thus it is suggested that more granular investigation be undertaken to better understand this usage pattern and where the beneficiaries may reside.

In general, it has been suggested that Latin American countries require more of a focus on national telemedicine policies and programs, including their regulatory frameworks and adequate funding [10]: a position that is supported by health system pandemic reviews [51]. However, from our narrative, it appears that Peru pre-empted this advice through its early and consistent advances on telemedicine legislation, strategies and plans over the pandemic’s preceding years. These advances appear to have given the country a quick entry for telemedicine and other digital health services when it was urgently required in an organized and structured manner. Peru’s response extends past patient-focused services to cover the delivery of online training services for health workers; a measure that helps to overcome some of telemedicine’s limitations [52]. During the pandemic, Peru trained 60% of their health workforce on skills and knowledge for the prevention and treatment of COVID-19, in a similar manner to other Latin American countries that ‘delivered mainly via distance learning technology or telemedicine platforms’ some of which ‘existed at health ministries prior to the pandemic’ [52, p.^27^].

However, despite Peru’s success with deployment, Fig. 2 shows that the number of telemedicine interactions fall-off as the pandemic progresses. We believe that there are a few possible reasons for this. Firstly, that telemedicine was only viewed as an emergency measure to reduce social distance and protect health workers under high Covid-19 infection rates [52]. Secondly, that the pattern of reducing utilization is reflective of the digital divide which exists in Peru driven by the country’s uneven access to ICT and technology skills in the population [46]. Thirdly, it is also possible that the pattern is influenced by factors in Latin America that impede telemedicine adoption. These have been identified as assuming that stakeholders will accept telemedicine and implementing telemedicine from a medical workforce perspective rather than focusing on the patients' needs, who’s poor engagement may be explained by low ICT skills [10]. Fourthly, there may be some health workforce or patient resistance to the continuation of telemedicine post-pandemic. Medical personnel tend to favour long-standing routines, have concerns about clinical quality, patient privacy and safety and worry about accountability should error occur [1], while patient preferences and experiences also affect their affinity to start or continue telemedicine consultations [11, 12]. Thus, these four possibilities lead us to suggest, along with other authors [36], that further research into effects and benefits of telemedicine for Peru throughout the pandemic is required. For without empirical evidence it is difficult to understand how an intervention works, to begin to evaluate alternatives, and to understand telemedicine’s cost-effectiveness to justify any continued investment and implementation [10]. In addition, there have been concerns raised of the quality and attractiveness of telemedicine to some sections of the population. For example, a study on Peru's millennials reveals that there are three main factors that affect this segment’s view of telemedicine: (1) Quality, where only 43% of interviewees perceive that the telemedicine service is of quality, (2) Immediacy, where only 42% consider telemedicine saves them time and (3) Personalization, where 70% would promote it if they found personalized plans according to their needs [12]. However, these results are not homogenous, as Fuentes and Navaro, 2021 also found the younger group of millennial patients, those between the ages of 26 and 34 years, consider that the quality of face-to-face care is superior to that of telemedicine and does not save time, while millennials between the ages of 34 and 41 years, married with children and mostly women, see telemedicine as of quality and saves them time [12]. Challenging these views of quality is a recent study of Peruvian cancer care during the pandemic that found 27.6% of the total cancer care delivered during the pandemic's first year was through telemedicine and that those patients’ satisfaction levels were 4.50 of maximum of 5.00 [53]. Together, such data reiterates the need for more fine-grained examinations of Peru's telemedicine usage statistics, patient use patterns and segment preferences to better understand uptake and the profiles of users.

These investigations could be undertaken by the Peruvian government or through partnerships with leading health, public policy and business technology universities. Such research is also valuable to strengthen the governance of telemedicine in Peru, as governance is considered a priority for improved telemedicine operations [10]. Thus, countries should have a focus on ensuring adequate regulatory instruments and change management strategies so that telemedicine services can become more a mainstream part of health services post pandemic. Peru appears to be responding, with telemedicine's inclusion in the National Health strategy and there are clear performance measures contained in its Digital Agenda for the Health Sector [35]. Although care needs to be taken in terms of continuing to include telemedicine as part of health financing and aligning these types of services with how future primary care models are to be developed to better serve Peru's health needs profile [29]. This includes the development of concurrent initiatives such as electronic patient records of which there is low access at primary care [36] and addressing the ongoing issues of the digital divide, health literacy, social traditions and technology skills, for both the health workforce and general population [37, 38]. So, while the newly resourced regional primary care facilities are likely to be afforded the opportunity to extend the reach of diagnostic and NCD monitoring services in Peru's remote and hard to reach areas it will be conditional on telephony and data network system and infrastructural restrictions [37, 38]. However, as Aguirre Martens notes, populations may have access to a health facility relatively close to their home, but after hours and weekend access maybe significantly restricted [31]. Thus, thought should also be given to how telemedicine care models more clearly align with primary care clinic programs, both to enhance local access and to address persistently high presentation rates at Peru's hospitals [29]. Moreover, the opportunity afforded by artificial intelligence is being also recognized for strengthening the nascent technologies and devices that are being trialled or within programs that are already operating that aim to improve diagnostic access in resource poor areas of Peru [54–56].

Thus, we can draw two lessons from the case of Peru. The first, as suggested by other authors, is that the country’s preparedness in terms of its telemedicine policy and regulation provided a platform for rapidly expanding digital health services when needed. That said there are some gaps, with the private medical app sector to address concerns regarding their security, privacy, information quality and effectiveness being identified, along with the quality of telemedicine services.

Secondly, that due to the pandemic, more patients and health care workers have had experiences with telemedicine. However, as the pandemic usage data show, as patient contact resumed telemedicine transactions significantly reduced. This phenomenon needs to be explored further to find the answers to what was driving this trend and how telemedicine transactions can be retained to continue to extend access, to provide care and disease monitoring and to reduce burdens on overstretched resources, particularly when patient preferences play a large part in continued telemedicine use [12]. Thus, Peru has a short-run window of opportunity to better understand and reinforce the benefits of telemedicine and importantly, to avoid its limitations to enable better access to services and to lift Peru’s UHC performance.

## CONCLUSION

In concurrence with the literature, we see those twenty years of the regulatory evolution of telemedicine in Peru prepared the country for its use in the pandemic. Its rapid rollout saw a significant growth of patient transactions early in the pandemic, though its use began to wane as the pandemic progressed and that Peru's telemedicine quality, attractiveness and access remain areas of concern.

As such, we identify two lessons from Peru's experiences, (1) that we reiterate that a comprehensive and thought through regulatory framework is necessary for quality telemedicine service deployment, and (2) that further research on the perspectives of health stakeholders regarding Peru's telemedicine pandemic experience is needed to provide contextual information to assist the country to normalize telemedicine as part of the health system and better align its health system with its health needs.

## Data Availability

The data underlying this article will be shared on reasonable request to the corresponding author.

## Footnotes

1 Using the average conversion rate of 3.6951 Nuevo Soles to the US dollar from February to August 2023.
